# Dataset on the structural characterization of organosolv lignin obtained from ensiled *Poaceae* grass and load-dependent molecular weight changes during thermoplastic processing

**DOI:** 10.1016/j.dib.2018.01.060

**Published:** 2018-02-01

**Authors:** Jörg Dörrstein, Ronja Scholz, Dominik Schwarz, Doris Schieder, Volker Sieber, Frank Walther, Cordt Zollfrank

**Affiliations:** a Chair for Biogenic Polymers, Technical University of Munich (TUM), Schulgasse 16, 94315 Straubing, Germany; b Department of Materials Test Engineering (WPT), TU Dortmund University, Baroper Str. 303, 44227 Dortmund, Germany; c Chair of Chemistry of Biogenic Resources, Technical University of Munich (TUM), Schulgasse 16, 94315 Straubing, Germany

## Abstract

This article presents experimental data of organosolv lignin from Poacea grass and structural changes after compounding and injection molding as presented in the research article “Effects of high-lignin-loading on thermal, mechanical, and morphological properties of bioplastic composites” [1]. It supplements the article with morphological (SEM), spectroscopic (^31^P NMR, FT-IR) and chromatographic (GPC, EA) data of the starting lignin as well as molar mass characteristics (mass average molar mass (M_w_) and Polydispersity (D)) of the extracted lignin. Refer to Schwarz et al. [2] for a detailed description of the production of the organosolv residue and for further information on the raw material used for lignin extraction. The dataset is made publicly available and can be useful for extended lignin research and critical analyzes.

**Specifications Table**

**Table:** 

Subject area	*Material Science*
More specific subject area	*Bioplastic composites, lignin research*
Type of data	*Tables, images, figures*
How data was acquired	*Scanning electron microscopy (SEM; DSM 940A, Zeiss, Germany), elemental analysis (EA; Euro EA, Hekatech, Germany), gel permeation chromatography (GPC; SECurtiy, PSS Polymer Standards Service, Germany) equipped with a refractive index detector and a series of linear columns (PSS Gram (30* *Å, 1000* *Å), AppliChrom ABOA DMSO-Phil-P (100* *Å)), High Performance Liquid Chromatography (HPLC; Dionex® equipped with a Rezex ROA-H+ column), 31 Phosphorous NMR (*^*31*^*P NMR; JEOL-ECS 400* *MHz, Jeol Ltd., Japan), Fourier-transform infrared spectroscopy (FT-IR; Nicolet 380, Thermo Scientific, Germany), image analysis (ImageJ, NIH, USA)*
Data format	*Raw spectra data, analyzed data, images*
Experimental factors	*The lignin-rich precipitate obtained from organosolvation of Poacea grass silage was Soxhlet extracted with ethyl acetate (EtOAc). The solid residue was used to fabricate lignin/polyethylene-co-vinyl acetate rubber composites. Thereafter, lignin was re-extracted with dimethyl sulfoxide (DMSO) from the bioplastic composites and structural data were collected*
Experimental features	*Structural data on lignin from ensiled Poaceae grass is given by solid- and liquid-state methods*
Data source location	*East Bavaria lower mountain range near Regensburg, Germany (49°14′N; 12°39′E)*
Data accessibility	*Data is available with this article*

**Value of the data**

● The data are convenient to examine the structural characteristics of organosolv lignin from herbaceous plants such as *Poaceae* grass and can be compared with other related studies.
● The data establish a link between lignin content in bioplastic composites and load-dependent molecular weight changes.
● These data allow other researchers to extend the characterization of lignin in highly-filled composites.

## 1 Data

**Fig. 1:**
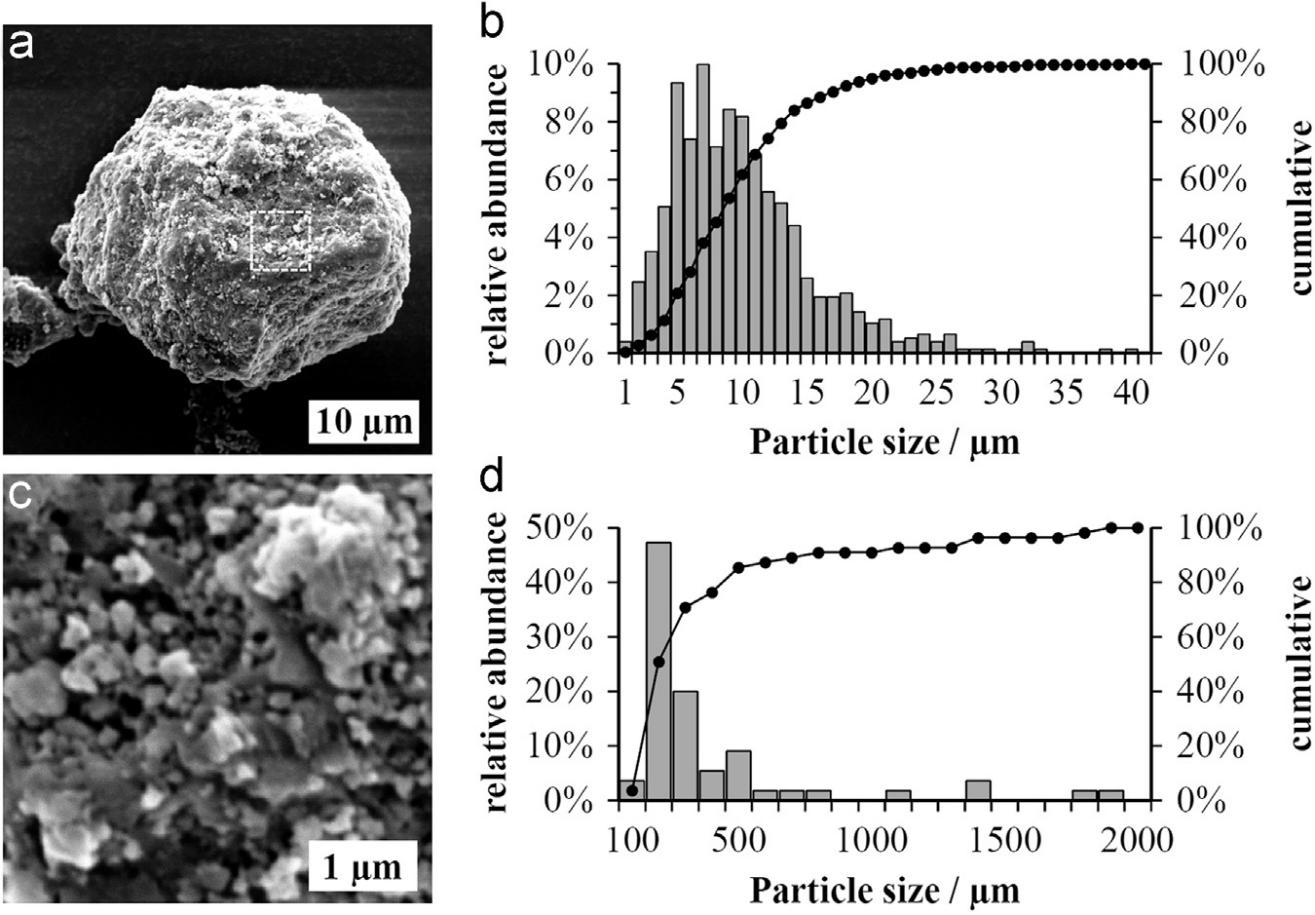
a) and c) Scanning electron micrographs of a precipitated *Poaceae* grass lignin particle displaying the particle surface and b) and d) size distribution of precipitated lignin particles and size distribution of clustered particles on the particle surface obtained from image analysis.

**Fig. 2:**
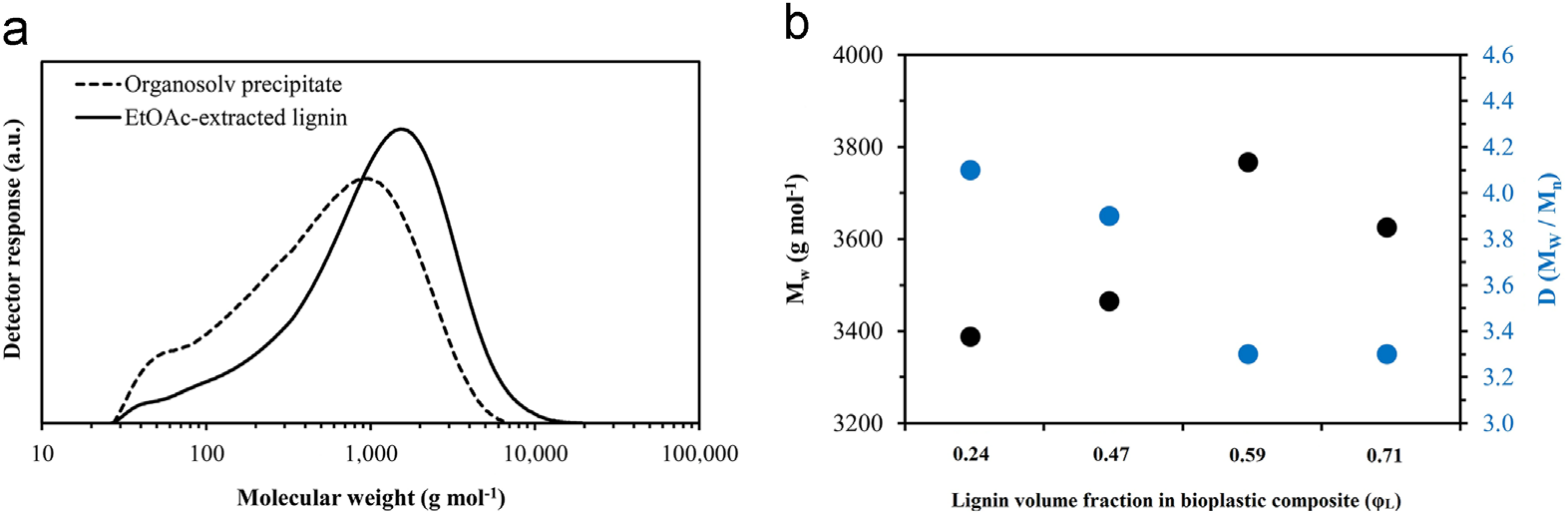
a) Molecular weight characteristics obtained from GPC of Organosolv precipitate and lignin after extraction with EtOAc. b) M_W_ and D of *Poaceae* grass lignin after injection molding and extraction with DMSO from bioplastic composites corresponding to different lignin volume fractions.

Morphological characteristics of lignin from *Poaceae* grass are shown in Fig. 1. Molecular weight change upon solvent-extraction of the organosolv residue and molecular weight changes of lignin as a function of lignin loading in processed lignin bioplastic composites [1]. are given in Fig. 2. Data on lignin purity are given in Table 1.

**Table 1:** Data on purity analysis of obtained *Poaceae* grass lignin: Mass average molecular weight, residual sugar, ash, sulfur content and mean particle size.

Molar mass		Purity			Mean particle size
*M*_w_	*D*	∑sugar	ash	sulfur	d_p_
(g mol^−1^)	(dimensionless)	(%)			(µm)
1600	3.3	3.0	1.1	0.1	9.5

**Fig. 3:**
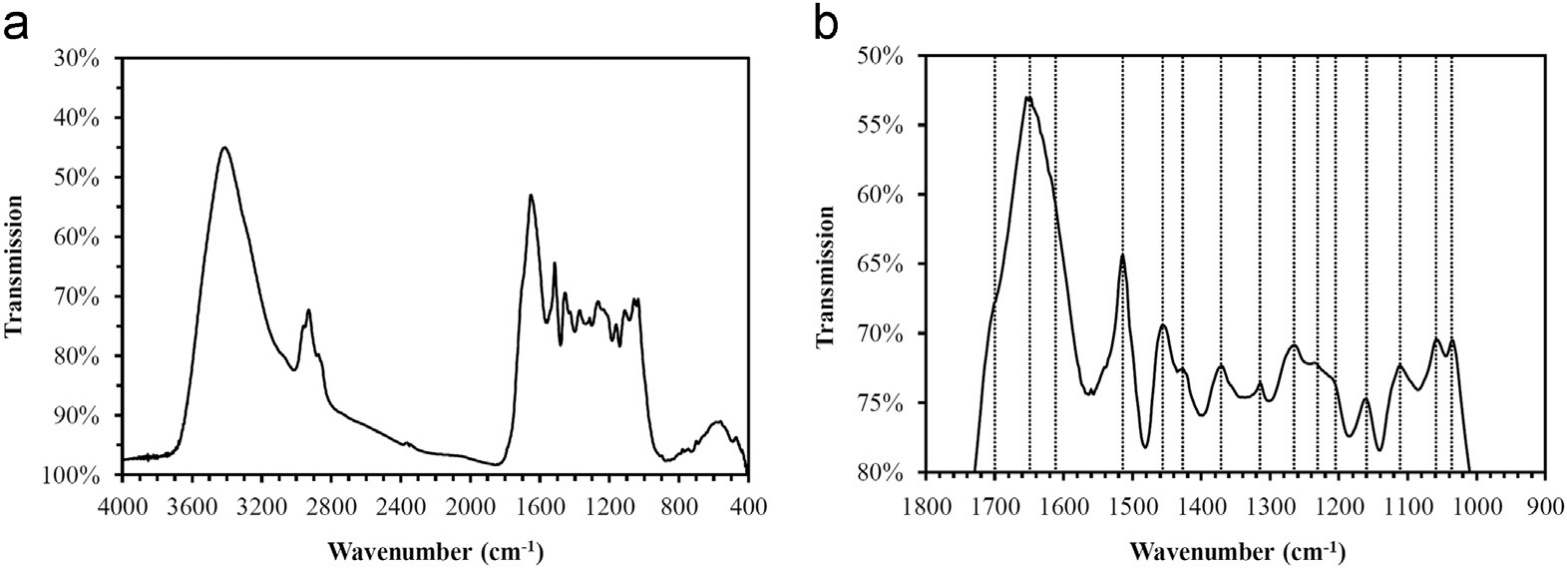
a) FT-IR spectrum of the isolated lignin (precipitated and EtOAc-extracted) and b) detail of the region below 1800 cm^−1^.

**Fig. 4:**
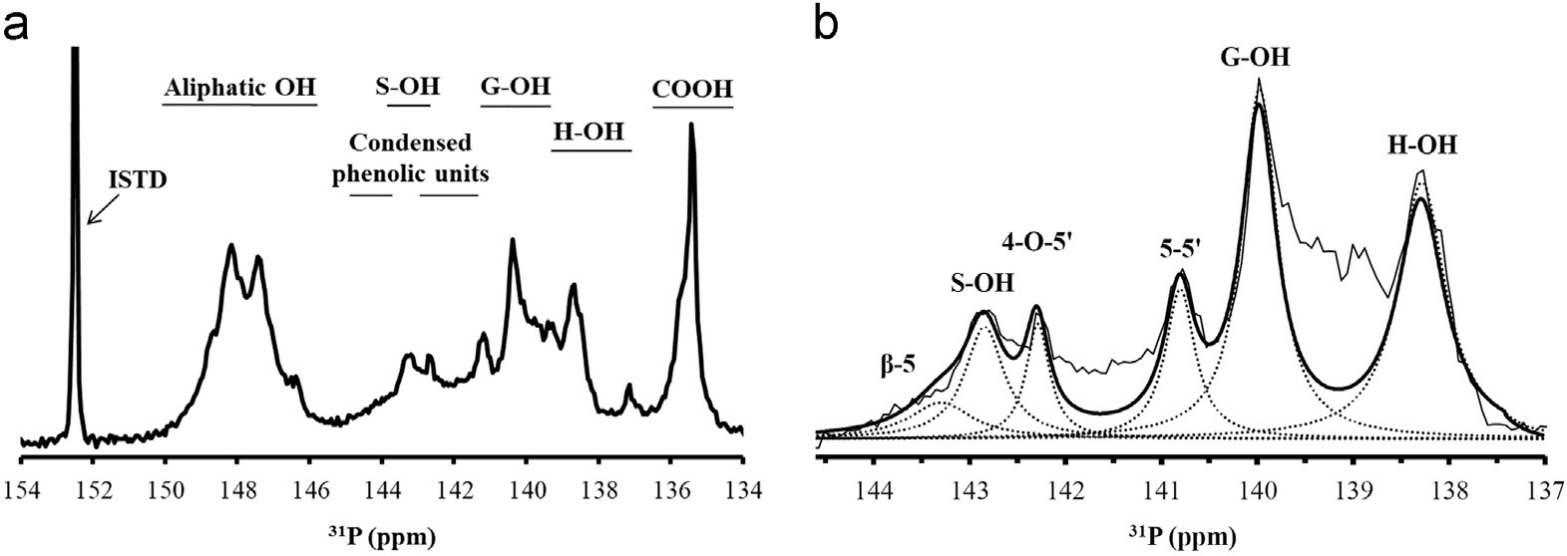
a) ^31^P NMR spectrum of phosphytylated starting lignin. b) ^31^P NMR spectra in the overlapping region between 144.5 and 137 ppm with deconvoluted signals.

Raw spectral data of the starting lignin are shown in Fig. 3, Fig. 4 and band assignments and hydroxyl group contents are given in Table 2, Table 3, respectively.

**Table 2:** FTIR band assignments of starting lignin.

**Wavenumber (cm**^**−1**^**)**	**Assignment**	**Reference**
1036	aromatic C-H in-plane deformation (G > S); C-O deform. in primary alcohols; C=O stretch (unconj.)	[3], [4], [5], [6]
1059	O–H stretch in cellulose	[4]
1111	guaiacyl C-H and syringyl C-H	[4]
1160	C=O stretch in conjugated ester groups, such as p-coumaric acid, typical for HGS lignins	[3], [5], [6], [7], [8]
1231	C-C stretch; C-O stretch; C=O stretch, G condensed > G etherified	[4], [8]
1265	C=O stretch; C-O stretch in guaiacyl aromatic methoxyl groups	[3], [4], [5], [6], [7], [8], [9]
~1315	condensed S and G ring (G ring substituted in pos. 5)	[3], [5], [6], [7], [8]
1371	aliphatic C-H stretch in CH_3_, not in OMe; phen. OH	[3], [5], [6], [7]
~1427	aromatic ring vibrations of phenyl-propane (C_9_) skeleton combined with C-H in-plane deformation	[3], [4], [5], [6], [7], [8], [9]
1456	C-H deformation; asym. in -CH_3_ and -CH_2_-	[3], [4], [5], [6], [7], [8], [9]
1514	aromatic skeleton vibrations (G > S)	[3], [4], [5], [6], [7], [8], [9]
~1612	aromatic skeletal vibrations (S > G); C=O stretch; G condensed > G etherified	[3], [4], [6]
1649	C=O stretch; in conjugated p-subst. aryl ketones; conjugated carbonyl and carboxyl; absorbed OH	[6], [8], [9], [10]
~1700	C=O stretch in unconjugated ketones, carbonyls and in ester groups; conjugated aldehydes and carboxylic acids absorb around and below 1700 cm^−1^	[3], [4], [5], [6], [7]
~2863	C-H vibration of mehtyl group of methoxyl	[3], [4], [5], [6], [7], [8], [9], [10]
2929	C-H stretch in -CH_3_ and -CH_2_-	[3], [4], [5], [6], [7], [8], [9], [10]
2964	C-H stretch in -CH_3_ and -CH_2_-	[3], [4], [5], [6], [7], [8], [9], [10]
3411	O-H stretch	[3], [4], [5], [6], [7], [8], [9], [10]

**Table 3:** Functional group contents obtained from quantitative ^31^P NMR where the assignments S-OH, G-OH, H-OH, COOH, 4-O-5′, 5-5′, and β-5 correspond to syringyl phenolic units, guaiacyl and demethylated phenolic units, p-hydroxylphenolic units, and carboxylic acids and condensed phenolic units of the 4-O-5’, 5-5’, and β-5 type.

Σ aliph. OH (mmol g^−1^)	Σ carboxyl. OH (mmol g^−1^)	Σ phenol. OH (mmol g^−1^)	β-5 (mmol g^−1^)	S-OH (mmol g^−1^)	4-O-5' (mmol g^−1^)	5-5'(mmol g^−1^)	G-OH (mmol g^−1^)	H-OH (mmol g^−1^)
1.49	0.31	0.59	0.06	0.10	0.07	0.11	0.25	0.23

## 2 Experimental design, materials and methods

### 2.1 Sample collection and preparation

A lignin-rich fraction was obtained by organosolvation of a grass silage press cake (PC) batch, described earlier by Schwarz et al. [2]. The obtained solid lignin phase was Soxhlet-extracted for 24 h using ethyl acetate (EtOAc), air dried overnight and then stored at ambient conditions until use.

### 2.2 Purity analysis

Lignin purity analysis was conducted according to NREL standard methods [11]. Acid insoluble lignin (Klason lignin) was examined by sulfuric acid hydrolysis. Residual carbohydrate and ash content were determined according to NREL/TP-510-48087 and sulfur content was determined using elemental analysis [12]. Measurements were run on vacuum-dried samples in duplicate and data are given as the arithmetic averages.

### 2.3 Fourier-transform infrared spectroscopy (FT-IR)

FT-IR analysis was performed to examine the starting lignin. Direct transmittance was measured by using the KBr pellet technique with a lignin concentration of 0.3 wt% in 300 mg KBr. The following parameters were used: spectral range: 400–4000 cm^−1^, spectral resolution: 2 cm^−1^, total scans: 128, background: KBr.

### 2.4 Morphological analysis

Mean lignin particle size and particle size distribution were evaluated using a scanning electron microscope operated at 10 kV and by image analysis.

### 2.5 ^31^P NMR

Spectral data were obtained according to a previously reported procedure and data on different functional groups present in lignin were obtained from integration of the spectra and calculated as described herein [13].

### 2.6 Gel permeation chromatography (GPC)

GPC was used to examine the mass average molecular weight and molecular weight distribution of isolated (starting) and processed lignin. For the determination of molecular weight changes following thermoplastic processing, lignin was Soxhlet-extracted for 24 h from ground composites using DMSO and lyophilized. The measurements were performed at 50 °C using 0.075 M DMSO/LiNO_3_ as the eluent. Lithium nitrate (LiNO_3_, anhydrous, 99.98%, Alfa Aesar, Germany) was added to minimize association effects. All samples were made up at 0.1% (w/v) in 0.075 M DMSO/LiNO_3_. Pullulan polymer standards (PSS) ranging from 180 to 708.000 g mol^−1^ were used for calibration.
