# Supplementary material for: Dataset on the structural characterization of organosolv lignin obtained from ensiled Poaceae grass and load-dependent molecular weight changes during thermoplastic processing
Source: Data Brief. 2018 Feb 1;17:647–52. doi: 10.1016/j.dib.2018.01.060 (PMC5852281; doi:10.1016/j.dib.2018.01.060)
Supplement: Supplementary file 1 — Supplementary material [file mmc1.docx]

The authors declare no conflict of interest.

Prof. Dr. Cordt Zollfrank

TUM – Chair for Biogenic Polymers
